# Prevalence of Metabolic Syndrome according to Sasang Constitutional Medicine in Korean Subjects

**DOI:** 10.1155/2012/646794

**Published:** 2012-02-09

**Authors:** Kwang Hoon Song, Sung-Gon Yu, Jong Yeol Kim

**Affiliations:** Division of Constitutional Medicine Research, Korea Institute of Oriental Medicine, 483 Expo-ro, Yuseong-gu, Daejeon 305-811, Republic of Korea

## Abstract

Metabolic syndrome (MS) is a complex disorder defined by a cluster of abdominal obesity, atherogenic dyslipidemia, hyperglycemia, and hypertension; the condition is recognized as a risk factor for diabetes and cardiovascular disease. This study assessed the effects of the Sasang constitution group (SCG) on the risk of MS in Korean subjects. We have analyzed 1,617 outpatients of Korean oriental medicine hospitals who were classified into three SCGs, So-Yang, So-Eum, and Tae-Eum. Significant differences were noted in the prevalence of MS and the frequencies of all MS risk factors among the three SCGs. The odds ratios for MS as determined via multiple logistic regression analysis were 2.004 for So-Yang and 4.521 for Tae-Eum compared with So-Eum. These results indicate that SCG may function as a significant risk factor of MS; comprehensive knowledge of Sasang constitutional medicine may prove helpful in predicting susceptibility and developing preventive care techniques for MS.

## 1. Introduction

Metabolic syndrome (MS) represents a cluster of metabolic risk factors which can be defined when three or more metabolic disorders are present, namely, high blood glucose, low high-density lipoprotein cholesterol (HDL-C), high blood pressure, high serum triglyceride (TG) levels, and abdominal obesity [1–4]. According to the Third National Health and Nutrition Examination Survey, the age-adjusted prevalence of MS as defined by the US National Cholesterol Education Program Adult Treatment Panel III (NCEP ATP III) is 23.7% in the general population but varies from 16 percent in black men to 37 percent in Hispanic women [5].

MS is significantly associated with increased risk of developing diabetes and cardiovascular disease (CVD). In particular, CVD is characteristically sudden in onset and life threatening; therefore it is necessary to develop a suitable preventive care method. Not only individuals with diabetes and CVD, but also individuals with MS are seemingly susceptible to other conditions, most notably polycystic ovary syndrome, fatty liver, cholesterol gallstones, asthma, sleep disturbances, and some forms of cancer [6]. It has been noted distinct patterns of cardiometabolic risk factors among different ethnic groups in the National Health and Nutrition Examination Survey (NHANES) sample [7, 8].

Sasang constitutional medicine (SCM) is a type of a Korean traditional medicine that classifies human beings into four constitutions, Tae-Yang (TY), So-Yang (SY), Tae-Eum (TE), and So-Eum (SE), based on their traits [9–11]. Each constitution shares common aspects of bodily structure, function, and metabolism, as well as psychological and behavioral characteristics [10]. Additionally, it has been indicated that each constitution has different susceptibility to pathology and the prevalence and relative risk of several chronic diseases were found to differ across different Sasang constitution groups (SCGs) [10, 12].

In SCM, the same disease is frequently treated differently for different individuals based on constitutionally differentiated traits in clinical practice [13]. Since MS is a significant precursor of diabetes and CVD, the constitutional differences in characteristics and prevalence of MS may facilitate improved patient care and prognoses. In this study, we assess the prevalence of MS and evaluate its risk factors in a large population-based study with Korean subjects classified by SCM.

## 2. Materials and Methods

### 2.1. Study Population

All samples and clinical information including his/her Sasang constitution type were deposited in the databank of Sasang constitutional medicine (DB-SCM) in Korea Institute of Oriental Medicine as previously described in detail [14]. However, 2 of the previous 1,619 participants were excluded due to the omission of their waist circumstance or blood pressure results. Therefore, the current study population included a total of 1,617 participants (593 males and 1,024 females).

### 2.2. Criteria for MS

MS was defined according to NECP ATP III guidelines, which stipulated that at least 3 out of 5 of the following criteria had to be met: (1) fasting blood glucose (FBG) ≥ 100 mg/dL or taking antihyperglycemic medication, (2) TG ≥ 150 mg/dL, (3) HDL-C ≤ 40 mg/d for male and 50 mg/dL for female, (4) systolic blood pressure (SBP) ≥ 130 mm Hg and/or diastolic blood pressure (DBP) ≥ 85 mm Hg or taking antihypertensive medication, and (5) central obesity with waist (WC) ≥ 90 cm for men and ≥80 cm for women. For FBG levels, we referred to American Diabetes Association (ADA) guidelines [15] and used a modified WC cutoff of ≥90 cm in men and ≥80 cm in women for abdominal obesity cutoff ranges [16].

### 2.3. Statistics

The quantitative variables were expressed as mean ± standard deviation. Student's *T*-test was used to analyze the difference between genders. Analysis of variance (ANOVA) was employed to analyze the differences among the SCGs. *χ* ^2^ tests were conducted to evaluate the significance of subgroup differences.

Multiple logistic regression analyses were conducted to calculate the odds ratio as a measure of relative risk of MS between SE and SY and between SE and TE. Odds ratios (ORs) were estimated with their 95% CI, and the significance threshold was set as *P* < 0.05. All statistical analyses were performed using SPSS (SPSS Inc., Chicago, IL; V14.0 for Windows).

## 3. Results

### 3.1. Characteristics and Prevalence of MS in the Total Study Population

A total of 1,617 individuals, 593 males and 1,024 females, were enrolled in this study. The general characteristics of study subjects, including age, FBG, HDL-C, SBP, DBP, TG, and WC, for this study as a total and according to gender were shown in Table 1.

The prevalence of MS and the relative frequencies of the individual risk factor of the MS in men and women are summarized in Table 2. Using the modified NECP ATP III diagnostic criteria, we detected a 35.37% prevalence of MS in the study population. Low HDL-C was the most common risk factor of MS according to the modified NECP ATP III criteria in both males and females. Males had a higher prevalence of hyperglycemia (*P* = 3.207*E* − 06), hypertension (*P* = 4.234*E* − 06), and hypertriglyceridemia (*P* = 2.911*E* − 09) than females. The frequency of large WC (*P* = 7.289*E* − 17) was higher in females than males. The frequency order of the risk factors of MS was low HDL-C > large WC > high BP > high FBG > high TG in total population, low HDL-C > high BP > high TG > large WC > high FBG in males, and large WC > low HDL-C > high BP > high FBG > high TG in females.

### 3.2. Prevalence of MS according to SCGs

To further address the prevalence of MS by SCM, 1,617 individuals were classified into three constitution groups and by gender, as described in Section 2. The clinical characteristics of the three SCGs separated by gender were described in Table 3. We detected statistically significant interconstitutional variations in all parameters.

The TE group evidenced significantly elevated FBG, SBP, DBP, TG, and WC and depressed HDL-C as compared to the SY and SE groups. The prevalence of MS was 18.02%, 30.59%, and 48.85% in total SE, SY, and TE, respectively (Table 4, *P* = 5.07*E* − 24). Both males and females evidenced significant differences in the prevalence of MS according to SCGs. Moreover, significant differences were noted in the frequencies of all MS risk factors among the three SCGs according to Chi-Square test results.

Low HDL-C was the top MS risk factor for MS using the modified NECP ATP III criteria in both SE and SY, but large WC was the top MS risk factor in TE. The frequency order of the risk factors of MS was as follows: low HDL-C > large WC > high BP > high FBG > high TG in SE and SY and large WC > low HDL-C > high BP > high TG > high FBG in TE. Interestingly, the frequencies of the individual risk factors of MS varied according to SCGs in both males and females. In males, the frequency order of the risk factors of MS was as follows: low HDL-C > high BP > high FBG > high TG > large WC in SE and SY and low HDL-C > large WC > high TG > high BP> high FBG in TE. In females, the frequency order of the risk factors of MS was as follows: low HDL-C > large WC > high BP > high FBG > high TG in SE, low HDL-C > large WC > high FBG > high BP > high TG in SY, and large WC > low HDL-C > high BP> high TG > high FBG in TE. High BP and high HDL-C were significantly more prevalent in the SE and SY than TE in males and females, respectively, but large WC was significantly more prevalent in the TE than SE and SY in both males and females.

**Figure 1:**
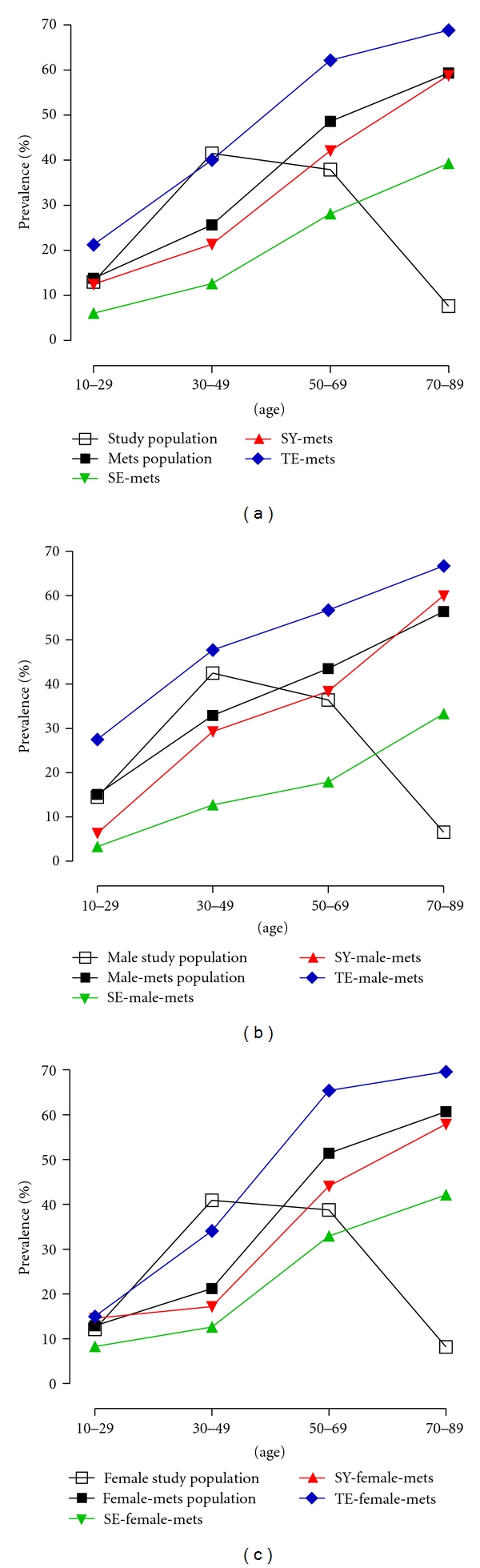
The prevalence of the MS according to SCG. The prevalence of MS according to SCG in total study population (a), male study population (b), and female study population (c) are shown, respectively.

The prevalence of MS by 20-year age groups is presented in Figure 1. The prevalence of MS increased with age in both males and females, with sharp increases at 30 S in males and at 50 S in females, respectively. Moreover, significance differences in the prevalence of MS were noted in each age category among the three SCGs via the Chi-Square test (*P* = 0.01, 8.412*E* − 11, 3.460*E* − 23, 2.974*E* − 05, resp.). The TE group evidenced the highest prevalence of MS, followed by SY and SE. However, there were no statistical significant in the prevalence of MS only in the 70–89 age category of males and in the 10–29 age category of females among three SCGs by the Chi-Square test.

### 3.3. ORs of MS according to SCGs

Since our findings indicated that SCG was a factor that was significantly associated with the prevalence of MS, we calculated ORs for MS using multiple logistic regression analysis to compare the prevalence of MS according to SCGs (Table 5). Using a logistic regression model with SE as a reference due to its lowest level of prevalence in MS, we determined that the ORs for occurrence of the MS were 2.004 for SY (95% CI, 1.467–2.738, *P* = 1.26*E* − 05) and 4.521 for the TE (95% CI, 3.364–6.074, *P* = 1.38*E* − 23). Additionally, significantly higher ORs were presented in males compared with females according to SCGs.

## 4. Discussion

The prevalence of MS has increased in recent years, particularly among older adults in Korea; this may constitute a public health problem [17]. The current study provided strong statistical support for the prevalence of MS and distinct risk profiles according to SCGs in Korea.

Although the prevalence of MS according to the modified NCEP ATP III criteria in the current study did not show gender-specific differences, the order of MS risk factors differed significantly according to gender. Consistent with the results of several population studies, we also reported an increase in the prevalence of MS with age suggesting that age-related changes in body size, fat distribution, and increases in insulin resistance contribute to the increased prevalence of MS with age [5, 17, 18]. Our data demonstrated that the males tend to have a higher prevalence of MS than the females in the 30–49 age group. However, the females have a higher prevalence than the males in the 50–69 age group, suggesting that menopause is one factor contributing to this change in MS prevalence [19, 20].

It should be noted that we observed the impact of SCG on the differences in the prevalence of MS and risk factor of MS. These findings are reminiscent of the theory of SCM, which holds that there are differences in susceptibility to pathological conditions according to SCG [10, 12]. It has been reported that the TE group tends to gain weight and that cardiovascular and metabolic disorders, such as hypertension, diabetes mellitus, and hyperlipidemia, appear with high prevalence in the TE group implying that TE is more predisposed to suffer from MS [10]. More interestingly, abdominal obesity predominated as a risk factor of MS in TE, but hypo-HDL-C predominated as a risk factor for MS in SE and SY.

Recently, we have reported that apolipoprotein A5-1131 genetic polymorphism is associated with lower serum HDL-C and higher TG in SY and TE, but not in SE subjects [14]. Interestingly, low HDL-C and high TG were more prevalent in SY and TE than in SE suggesting that the association of this polymorphism with MS is attributable to its effects on HDL-C and TG metabolism. However, the genetic basis of MS according to SCG remains to be definitively elucidated.

In our study, SCG was associated significantly with increased odds of MS in our multiple logistic regression analysis. In agreement with previous reports that the prevalence of diabetes and insulin resistance were higher in TE than in SE and SY [12, 21], we found that the prevalence of MS was higher in TE than in SE and SY. Furthermore, the ORs of MS were also higher with age in TE (See Supplementary Table S1 available online at doi: 10.1155/2012/646794).

The differences in the prevalence of MS according to SCG suggest that SCG may function as a significant risk factor of MS susceptibility [10]. The present study not only discloses the exceptionally higher prevalence of MS in TE than in SE and SY, but also suggests the beginnings of a comprehensive understanding of SCG for preventive SCG-specific care. Additionally, the number of participants with one or more MS risk factors suggests the TE group is potentially at risk for developing MS (See Supplementary Table S2). Therefore, we believe that the comprehensive knowledge of SCG may prove helpful in predicting MS susceptibility; our findings should attract increased attention to the typological aspects of classifying a very high portion of the asymptomatic population such as TE, as being in need of counseling, and overall risk assessment for metabolic diseases such as CVD.

## 5. Conclusions

In conclusion, the results of this study demonstrated that the prevalence of MS and the frequencies of all MS risk factors among the three SCGs; the TE group evidenced the highest prevalence of MS followed by the SY and SE groups. These results indicate that SCG may function as a significant risk factor for MS, and comprehensive knowledge of SCM may prove helpful in predicting susceptibility to and adequate methods for the preventive care of MS.

## Supplementary Material

Supplementary Table1: Odds ratio of MS according to age and SCG.
Supplementary Table2: The distribution of MS risk factor holder according to gender and SCG.

## Figures and Tables

**Table 1:** General characteristics of the study subjects.

Variables	Mean ± SD			*P* value
	Male (*n* = 593)	Female (*n* = 1024)	Total (*n* = 1617)	
Age (years)	47.26 ± 14.91	48.45 ± 14.95	48.12 ± 14.9	0.078
FBG (mg/dL)	102.17 ± 30.48	96.45 ± 25.23	98.56 ± 27.53	**5.81** **E** − 05
HDL-C (mg/dL)	41.7 ± 10.20	48.66 ± 11.98	46.11 ± 11.83	**2.52*E* − 31**
SBP (mmHG)	122.99 ± 13.88	119.08 ± 14.94	120.52 ± 14.67	**2.25*E* − 07**
DBP (mmHG)	79.68 ± 10.55	76.28 ± 11.17	77.53 ± 11.07	**2.37*E* − 09**
TG (mg/dL)	150.52 ± 97.26	117.48 ± 72.61	129.7 ± 83.84	**1.79*E* − 14**
WC (cm)	86.96 ± 8.86	82.7 ± 10.09	84.27 ± 9.87	**4.01*E* − 17**

Values are indicated as the mean±standard deviation.

*P* value: Student's *T*-test result between male and female.

FBG: fasting blood glucose; HDL-C: high-density lipoprotein cholesterol; SBP: systolic blood pressure; DBP: diastolic blood pressure; TG: triglyceride; WC: waist circumference.

**Table 2:** The prevalence of MS and risk factors of the study subjects.

Variables		Number (%)		*P* value
	Male	Female	Total	
MS	212 (35.75)	360 (35.16)	572 (35.37)	0.810
High FBG	220 (37.1)	267 (26.07)	487 (30.12)	**3.207*E* − 06**
Low HDL-C	283 (47.72)	596 (58.2)	879 (54.36)	**4.559*E* − 05**
High BP	242 (40.81)	303 (29.59)	545 (33.70)	**4.234*E* − 06**
High TG	223 (37.61)	243 (23.73)	466 (28.82)	**2.911*E* − 09**
Large WC	221 (37.27)	602 (58.79)	823 (50.9)	**7.289*E* − 17**

Values are indicated as number (%).

*P* value: Chi-square test between male and female.

MS: metabolic syndrome; FBG: fasting blood glucose; HDL-C: high-density lipoprotein cholesterol; TG: triglyceride; WC: waist circumference.

**Table 3:** General characteristics of the study subjects according to SCG.

		SE			SY			TE		*P* value^a^	*P* value^b^	*P* value^c^
Variables	Male	Female	Total	Male	Female	Total	Male	Female	Total			
	(*n* = 141)	(*n* = 264)	(*n* = 405)	(*n* = 186)	(*n* = 360)	(*n* = 546)	(*n* = 266)	(*n* = 400)	(*n* = 666)			
Age (years)	44.14 ± 15.89	46.65 ± 14.63	45.77 ± 15.11	49.39 ± 14.07	46.91 ± 14.65	47.76 ± 14.49	47.42 ± 14.72	51.44 ± 14.86	49.83 ± 14.92	**0.007**	**6.31*E* − 06**	**6.51*E* − 05**
FBG (mg/dL)	97.09 ± 25.15	91.40 ± 13.40	93.38 ± 18.53	104.25 ± 31.39	96.46 ± 28.72	99.11 ± 29.86	103.41 ± 32.18	99.82 ± 27.79	101.25 ± 29.65	0.073	**1.56*E* − 04**	**2.68*E *− 05**
HDL-C (mg/dL)	45.05 ± 10.09	51.34 ± 11.99	49.15 ± 11.74	41.81 ± 10.81	49.21 ± 11.78	46.69 ± 11.98	39.85 ± 9.37	46.41 ± 11.69	43.79 ± 11.29	**5.07*E* − 06**	**6.39*E* − 07**	**1.44*E* − 12**
SBP (mmHG)	119.1 ± 13.38	115.14 ± 14.1	116.52 ± 13.97	122.07 ± 13.63	117.41 ± 13.45	118.99 ± 13.68	125.7 ± 13.78	123.21 ± 15.76	124.21 ± 15.04	**1.40*E* − 05**	**1.37*E* − 12**	**4.89*E* − 18**
DBP (mmHG)	77.41 ± 11.85	73.85 ± 11.08	75.09 ± 11.47	79.92 ± 10.37	75.19 ± 10.37	76.8 ± 10.6	80.71 ± 9.79	78.89 ± 11.42	79.62 ± 10.83	**0.01**	**4.89*E* − 09**	**8.78*E* − 11**
TG (mg/dL)	111.72 ± 58.15	96.50 ± 49.36	101.80 ± 53.02	158.58 ± 104.80	114.10 ± 67.92	129.25 ± 84.93	165.45 ± 103.00	134.77 ± 84.02	147.02 ± 93.22	**2.19*E* − 07**	**6.90*E* − 11**	**5.40*E* − 17**
WC (cm)	80.47 ± 8.43	77.65 ± 9.11	78.64 ± 8.97	86.02 ± 6.90	80.14 ± 8.74	82.16 ± 8.62	91.11 ± 8.04	88.32 ± 9.12	89.44 ± 8.81	**9.24*E* − 34**	**8.39*E* − 52**	**5.52*E* − 82**

Values are indicated as the mean ± standard deviation.

*P* value^a^: Analysis of variance (ANOVA) result of male among SCG.

*P* value^b^: Analysis of variance (ANOVA) result of female among SCG.

*P* value^c^: Analysis of variance (ANOVA) result of total among SCG.

SY: So-Yang; SE: So-Eum; TE: Tae-Eum; FBG: fasting blood glucose; HDL-C: high-density lipoprotein cholesterol; SBP: systolic blood pressure; DBP: diastolic blood pressure; TG: triglyceride; WC: waist circumference.

**Table 4:** The prevalence of MS and risk factors according to SCG.

Variables		SE			SY			TE		*P* value^a^	*P* value^b^	*P* value^c^
	Male	Female	Total	Male	Female	Total	Male	Female	Total			
MS	19 (13.48)	54 (20.45)	73 (18.02)	62 (33.33)	105 (29.17)	167 (30.59)	131 (49.25)	201 (50.25)	332 (48.85)	**5.03*E* − 12**	**4.51*E* − 16**	**5.07*E* − 24**
High FBG	37 (26.24)	48 (18.18)	85 (20.99)	77 (41.40)	92 (25.56)	169 (30.95)	106 (39.85)	127 (31.75)	233 (34.98)	**0.009**	**4.84*E* − 04**	**7.08*E* − 06**
Low HDL-C	46 (32.62)	130 (49.24)	176 (43.46)	86 (46.24)	205 (56.94)	291 (53.30)	151 (56.77)	261 (65.25)	412 (61.86)	**1.87*E* − 05**	**1.92*E* − 04**	**2.83*E* − 08**
High BP	40 (28.37)	50 (18.94)	90 (22.22)	84 (45.16)	86 (23.89)	170 (31.14)	118 (44.36)	167 (41.75)	285 (42.79)	**0.002**	**3.12*E* − 11**	**1.30*E* − 11**
High TG	25 (17.73)	40 (15.15)	65 (16.05)	74 (39.78)	73 (20.28)	147 (26.92)	124 (46.62)	130 (32.50)	254 (38.14)	**5.82*E* − 08**	**2.91*E* − 07**	**4.77*E* − 14**
Large WC	17 (12.06)	101 (38.26)	118 (29.14)	54 (29.03)	179 (49.72)	233 (42.67)	150 (56.39)	322 (80.50)	472 (70.87)	**2.95*E* − 19**	**2.98*E* − 30**	**1.10*E* − 43**

Values are indicated as number (%).

*P* value^a^: Chi-square test of male among SCG.

*P* value^b^: Chi-square test of female among SCG.

*P* value^c^: Chi-square test of total among SCG.

MS: metabolic syndrome; SY: So-Yang; SE: So-Eum; TE: Tae-Eum; FBG: fasting blood glucose; HDL-C: high-density lipoprotein cholesterol; TG: triglyceride; WC: waist circumference.

**Table 5:** Odds ratio of MS according to SCG.

Variables	Gender	Constitution (Number)	OR	95% CI		*P*-value
				Lower	Upper	
MS	Total	SE (405)	1	—	—	—
		SY (546)	**2.004**	1.467	2.738	**1.26*E* – 05**
		TE (666)	**4.521**	3.364	6.074	**1.38*E* – 23**
	Male	SE (141)	1	—	—	—
		SY (186)	**3.211**	1.813	5.686	**6.33*E ***− **05**
		TE (266)	**6.231**	3.631	10.691	**3.09*E *** − ** 11**
	Female	SE (264)	1	—	—	—
		SY (360)	**1.601**	1.1	2.331	**0.014**
		TE (400)	**3.928**	2.747	5.616	**6.41*E *** − ** 14**

Abbreviations: MS, metabolic syndrome; SY, So-Yang; SE, So-Eum; TE, Tae-Eum; OR, odds ratio; CI, confidence interval.

*P*-value: multiple logistic regression analysis according to SCG.

Numbers of participants are indicated in a round bracket.

## Abbreviations

MS: Metabolic syndrome
SCM: Sasang constitutional medicine
SCG: Sasang constitution group
SE: So-Eum
SY: So-Yang
TE: Tae-Eum
TG: Triglyceride
FBG: Fasting blood glucose
HDL-C: High-density lipoprotein cholesterol
SBP: Systolic blood pressure
DBP: Diastolic blood pressure
WC: Central obesity with waist.
