# Supplementary material for: Prevalence of Metabolic Syndrome according to Sasang Constitutional Medicine in Korean Subjects
Source: Evid Based Complement Alternat Med. 2012 Feb 9;2012:646794. doi: 10.1155/2012/646794 (PMC3290894; doi:10.1155/2012/646794)
Supplement: Supplementary file 1 — Supplementary Table1: Odds ratio of MS according to age and SCG. Supplementary Table2: The distribution of MS risk factor holder according to gender and SCG. [file 646794.f1.pdf]

Supplementary Table 1. Odds ratio of MS according to SCG

Variables	Gender	Age	Constitution	OR	95% CI		*P*-value
					Lower	Upper	
MS	Total	10-29	SE	1	-	-	
			SY	2.21	0.63	7.76	0.214
			TE	**4.18**	1.32	13.13	**0.014**
		30-49	SE	1	-	-	-
			SY	**1.88**	1.11	3.18	**0.02**
			TE	**4.63**	2.81	7.62	**1.816E-09**
		50-69	SE	1	-	-	-
			SY	1.86	1.15	3.01	0.12
			TE	**4.21**	2.65	6.69	**1.225E-09**
		70-89	SE	1	-	-	-
			SY	2.21	0.80	6.13	0.128
			TE	**3.42**	1.35	8.68	**0.01**
	Male	10-29	SE	1	-	-	-
			SY	1.93	0.11	33.13	0.649
			TE	**11.00**	1.33	90.81	**0.026**
		30-49	SE	1	-	-	-
			SY	**2.85**	1.18	6.87	**0.02**
			TE	**6.26**	2.72	14.40	**1.590E-05**
		50-69	SE	1	-	-	-
			SY	**2.84**	1.11	7.31	**0.03**
			TE	**5.99**	2.42	14.82	**1.055E-04**
		70-89	SE	1	-	-	-
			SY	3.00	0.53	16.90	0.213
			TE	4.00	0.69	23.09	0.121
	Female	10-29	SE	1	-	-	-
			SY	1.88	0.45	7.83	0.387
			TE	1.94	0.45	8.41	0.375
		30-49	SE	1	-	-	-
			SY	1.44	0.74	2.81	0.284
			TE	**3.59**	1.90	6.76	**7.800E-05**
		50-69	SE	1	-	-	-
			SY	1.61	0.91	2.85	0.103
			TE	**3.84**	2.21	6.69	**1.899E-06**
		70-89	SE	1	-	-	-
			SY	1.89	0.52	6.85	0.332
			TE	**3.14**	1.04	9.50	**0.042**

Abbreviations: MS, metabolic syndrome; SY, So-Yang; SE, So-Eum; TE, Tae-Eum; OR, odds ratio; CI, confidence interval

P-value: multiple logistic regression analysis according to SCG

Supplementary Table 2. The distribution of MS risk factor holder according to gender and SCG

Number of risk factor	Number (%)					
	Male	Female	Total	SE	SY	TE
0	96 (19.19)	172 (16.80)	268 (16.57)	124 (30.62)	91 (16.67)	53 (7.96)
1	132 (22.26)	250 (24.41)	382 (23.62)	121 (29.88)	150 (27.47)	111 (16.67)
2	153 (25.8)	242 (23.63)	395 (24.43)	87 (21.48)	138 (25.27)	170 (25.53)
3	118 (19.9)	211 (20.61)	329 (20.35)	55 (13.58)	103 (18.86)	171 (25.68)
4	73 (12.31)	101 (9.86)	174 (10.76)	16 (3.95)	45(8.24)	113 (16.97)
5	21 (3.54)	48 (4.69)	69 (4.27)	2 (0.49)	19 (3.48)	48 (7.21)

Values are indicated as number (%)

Abbreviations: SE, So-Eum; SY, So-Yang; TE, Tae-Eum
